# Urban nature at the fingertips: Investigating wild food foraging to enable nature interactions of urban dwellers

**DOI:** 10.1007/s13280-021-01648-1

**Published:** 2021-10-28

**Authors:** Christoph Schunko, Anjoulie Brandner

**Affiliations:** University of Natural Resources and Life Sciences, Vienna (BOKU), Gregor-Mendel-Strasse 33, 1180 Vienna, Austria

**Keywords:** Edible city, Nature connectedness, Provisioning ecosystem services, Urban food forestry, Urban non-timber forest product, Wild plant gathering

## Abstract

Meaningful human–nature interactions can counteract the extinction of experience and positively influence people’s nature relatedness, health and wellbeing. In this study, we explored urban wild food foraging to understand how best to enable human-nature interactions in cities by means of foraging. Using a structured questionnaire, a total of 458 residents of Vienna, Austria were surveyed. Sixty-four percent of visitors of public urban green spaces previously foraged for wild food species, whereas foraging frequencies were related to the targeted plant species and their life forms. People who foraged more frequently had greater nature relatedness, more childhood foraging experiences and lived on the outskirts of the city, but their socio-demographic backgrounds were heterogeneous. Social acceptance and lack of access to wild foods were perceived to be barriers. To promote nature relatedness through urban foraging, the legal framework, access to low-contamination foraging areas, availability of wild foods and social acceptance need to be improved.

**Supplementary Information:**

The online version contains supplementary material available at 10.1007/s13280-021-01648-1.

## Introduction

Urban nature, such as street trees, parks and gardens, has many positive effects on urban dwellers, reducing their stress levels and increasing social cohesion and physical activity (Hartig et al. 2014; Hartig and Kahn 2016). These benefits can be further enhanced when urban dwellers get in meaningful emotional and sensorial contact with urban nature (Lumber et al. 2017; Colléony et al. 2020). This direct interaction with nature can also enhance nature relatedness, the subjective connection individuals have with nature (Nisbet and Zelenski 2013), lead to further positive impacts on health and wellbeing, including happiness (Capaldi et al. 2014), and stimulate pro-environmental behaviour (Colléony et al. 2020; Martin et al. 2020). There has been a continuous fall in direct human–nature interactions for decades, a trend termed extinction of experience (Soga and Gaston 2016). One strategy to counteract this trend is to engage urban dwellers in urban food production (Lin et al. 2018; Kingsley et al. 2021). Related initiatives have evolved quickly in recent years, a development reflected in the range of buzzwords such as edible cities (Sartison and Artmann 2020), edible landscapes (McLain et al. 2012), edible green infrastructure (Russo et al. 2017), edible urban commons (Sardeshpande et al. 2021), urban gardening (Coles and Costa 2018) and urban food forestry (Clark and Nicholas 2013). In addition, the global COVID-19 pandemic has further raised awareness of the need to regionalise food production, which will arguably boost initiatives targeting urban food production (Sardeshpande et al. 2021).

Urban foraging for wild foods, conceptualised as picking, pinching or gathering edible organs of plants and mushrooms that grow without intentional tending and cultivation for food production in cities (adapted from Poe et al. (2013)), is a related activity that has received relatively little attention (Shackleton et al. 2017). However, urban foraging is particularly well-suited to promoting meaningful human-nature interactions in cities because it allows spontaneous and sporadic interactions and appeals equally to a wide range of urban dwellers, irrespective of their sex, age, education, income (Gianotti and Hurley 2016; Fischer and Kowarik 2020) or cultural and ethnic backgrounds (Pieroni et al. 2005; McLain et al. 2014). While urban dwellers’ socio-demographic background plays no part in urban foraging, urban foragers depend on the accessibility of foraging locations. Therefore, foraging is more widespread among urban dwellers living on the outskirts, where more green space is available, than in densely built-up areas in city centres (Schlesinger et al. 2015; Gianotti and Hurley 2016; Somesh et al. 2021). In addition, those households with access to private gardens tend to forage less in public urban green spaces (Kaoma and Shackleton 2014; Mollee et al. 2017), while some urban foragers travel significant distances to forage for more varieties and greater quantities of plant materials (Synk et al. 2017).

The foraged wild food taxa include a wide range of different species, including woody and herbaceous, native and introduced, and abundant and rare species (Palliwoda et al. 2017; Landor-Yamagata et al. 2018; Fischer and Kowarik 2020; Somesh et al. 2021). Most urban foragers gather a few well-known and abundant taxa that are easy to identify and forage (Synk et al. 2017; Fischer and Kowarik 2020). Nevertheless, some experienced foragers gather large numbers of different taxa (McLain et al. 2014; Fischer and Kowarik 2020), including rare ones (Landor-Yamagata et al. 2018). For example, experienced urban foragers in Baltimore forage for 170 different wild food taxa (Synk et al. 2017) and in Berlin for 125 different ones (Landor-Yamagata et al. 2018). Urban foraging is a recreational side-activity for most foragers, whereas dedicated urban foragers in Baltimore forage up to once a week and for more than 10% of their daily food needs (Synk et al. 2017). These numbers highlight the varying significance of urban foraging to urban dwellers, but urban foraging has untapped potential. In New York City, 201 woody species—66% of all woody species growing in the city—have edible plant parts (Hurley and Emery 2018), and in Burlington, Vermont, the entire population could be supplied with the recommended daily quantities of fruit if a significant share of public urban green spaces were planted with fruit trees (Clark and Nicholas 2013).

In cities where urban foraging has been studied, it is largely unsupported politically and therefore several barriers are commonly found (Shackleton et al. 2017). First, public authorities tend to be ambivalent about urban foraging, and in most cases the legal frameworks either do not regulate the activity at all or they restrict it (McLain et al. 2014; Shackleton et al. 2017). The main concern raised by public authorities is that urban foraging damages urban vegetation (McLain et al. 2014). Indeed, urban foragers sometimes forage for endangered plant species (Landor-Yamagata et al. 2018) or large quantities of plants (McLain et al. 2017), and urban foragers themselves sometimes express doubts about the foraging practices of other urban dwellers (Charnley et al. 2018). However, these observations are counterbalanced by a number of stewardship practices performed by urban foragers that contribute to maintaining and even enhancing urban vegetation, including rotating foraging areas, undertaking selective foraging, transplanting or spreading seeds, removing invasive species and collecting rubbish (McLain et al. 2017; Schunko et al. 2021). Such stewardship practices are acknowledged in part by public authorities, but concerns about urban vegetation remain (Sardeshpande and Shackleton 2020). In addition, urban foragers also face the challenge of informal regulations that impede access to the large number of different urban green spaces for foraging (Charnley et al. 2018). A major barrier is also the potential contamination of urban wild foods by pollution, traffic or dog excrement, which affects the safety of consuming those foods (Mollee et al. 2017; Fischer and Kowarik 2020). Other barriers are the lack of availability of urban green spaces where wild foods can be foraged (Garekae and Shackleton 2020; Somesh et al. 2021) and very limited knowledge about plant identification, foraging locations or foraging practices (Gaither et al. 2020).

With this study, we aimed to gain insights into urban foraging to inform urban planning strategies that intend to provide urban dwellers with more opportunities to interact with urban nature. More precisely, we aimed to understand: How widespread is urban wild food foraging? Which wild food species and plant life forms are preferred by urban foragers? What characteristics of urban dwellers explain their urban foraging frequencies? What barriers to urban wild food foraging do urban foragers perceive? This study focused on foraging in public urban green spaces rather than on private land and adopted a similar approach to that taken in several broad-based and recently published papers about urban foraging in cities in Africa (Mollee et al. 2017; Garekae and Shackleton 2020), Asia (Somesh et al. 2021), Europe (Landor-Yamagata et al. 2018; Fischer and Kowarik 2020) and North America (McLain et al. 2013; Gianotti and Hurley 2016; Synk et al. 2017; Gaither et al. 2020). Building on the methods used and insights gained from these papers, we adopted methodological and content-related innovations by (i) comparing the foraging frequencies of urban dwellers in urban spaces versus rural spaces to better understand the peculiarities of urban foraging; (ii) using a validated nature-relatedness scale to test if the suggested association with urban foraging holds true; and (iii) measuring urban dwellers’ knowledge and reported behaviour about preventing negative ecological impacts when foraging to assess the risk of urban foraging to vegetation.

## Materials and methods

### Field site

This study took place in Vienna, which has a population of 1.9 million and is the largest city and capital of Austria. Almost half of the city’s area consists of urban green spaces (18 990 ha out of a total of 41 487 ha), of which two thirds (12 600 ha) are publicly accessible (Stadt Wien 2019). The most widespread public urban green spaces are forests and meadows (73%), followed by parks (19%) and agricultural landscapes (4%) (MA22 2021). In Vienna, more than two thirds of residents have access to an urban green space within 500 m of their home (Kabisch et al. 2016). Wild food foraging is popular in Austria, with one study indicating that 89% of almost 500 organic consumers in different parts of the country forage (Schunko and Vogl 2020). The legal framework for foraging in Vienna is fragmented across a number of different regulations and laws. Urban foraging tends to be prohibited in intensively managed city centre parks and nature reserves, but is allowed for personal use in less managed public urban green spaces on the city’s outskirts.

### Sample

We surveyed urban residents using two different sampling strategies. The aim with the first sub-sample was to survey the breadth of Vienna’s population, conducting face-to-face surveys in eight public urban green spaces of different sizes and centralities. Each selected public urban green space was visited three to four times on different days of the week and times of the day. We stayed on the main paths and entrances and approached every visitor we encountered. Between 28 and 31 urban residents were interviewed at each public urban green space, giving a total of 236 altogether. With the second sub-sample, the aim was to survey urban dwellers who were experienced in urban foraging. Therefore, we created an online survey and asked urban foragers, environmental educators and representatives of research institutions and non-governmental institutions working on urban foraging to send out a link to the online survey via their websites, newsletters and mailing lists. We also posted the link to the survey on social networking platforms related to wild plants, foraging, plant sharing and seed exchange. Altogether, 222 urban residents responded to the online survey. In both sub-samples, the respondents had to be resident in Vienna. Taking both samples together, 69% of the respondents were female, 59% had been to university, 38% lived in Vienna’s central districts, 61% had spent their childhood in a city and 47% had access to a private garden. The mean age of respondents was 41 years (SD = 16) and the mean duration of residence in Vienna was 26 years (SD = 20) (Table 1).

**Table 1:** Summary statistics and explanations of predictor and outcome variables used in multiple linear regressions

Variables	Explanation	Total (entire sample)			Face-to-face survey (sub-sample 1)			Online survey (sub-sample 2)		
Predictor variables		*n*	Mean*	SD*	*n*	Mean	SD	*n*	Mean	SD
Sex	1 = female, 2 = male	444	1.31	0.5	230	1.43	0.5	214	1.18	0.4
Age	Age in years	451	41.2	16.2	230	45.7	17.9	221	36.5	12.6
Higher education	Graduated from a university (1 = No, 2 = Yes)	458	1.59	0.5	236	1.45	0.5	222	1.75	0.4
Residence time in Vienna	Number of years residing in Vienna	453	25.6	19.6	231	30.4	21.2	222	20.5	16.3
Residence in central districts	Residence in a heavily built-up central district of Vienna with a low percentage of public urban green spaces (Districts 1, 3–9, 12, 15, 20) (1 = No, 2 = Yes)	456	1.38	0.5	234	1.38	0.5	222	1.37	0.5
Access to a private garden	Access to a private garden (1 = No, 2 = Yes)	458	1.47	0.5	236	1.42	0.5	222	1.54	0.5
Childhood in a city	Residence in a city of more than 10 000 inhabitants before the age of 16 (1 = No, 2 = Yes)	458	1.61	0.5	236	1.67	0.5	222	1.55	0.5
Childhood foraging	Frequency of foraging for wild foods before the age of 16 (1 = < 1 time/year; 2 = 1–3 times/year; 3 = > 3 times/year	458	2.14	0.8	236	2.15	0.8	222	2.13	0.8
Nature relatedness	Mean agreement with 6 items on 5-step nature relatedness scale	458	4.00	0.8	236	4.11	0.7	222	3.89	0.8
Set number	Set of wild food species shown to respondents in interviews Set 1 = *Allium ursinum* L., *Fragaria* sp., *Tilia* sp., *Prunus domestica* subsp. *insititia* (L.) Bonnier & Layens Set 2 = *Urtica* sp., *Rubus fruticosus* agg., *Sambucus nigra* L., *Juglans regia* L	458			236			222		
Outcome variables										
Rural foraging frequency	Foraging frequency of four plant species (set 1 or 2) in the countryside outside of private gardens in the last 5 years (4-point Likert scale: 0 points = never, 0.33 points = less frequently than every 2–3 years, 0.66 points = every 2–3 years, 1 point = (almost) every year)	458	1.35	1.2	236	0.85	1.0	222	1.87	1.2
Urban foraging frequency	Foraging frequency of four plant species (set 1 or 2) in Vienna outside of private gardens in the last 5 years (4-point Likert scale: 0 points = never, 0.33 points = less frequently than every 2–3 years, 0.66 points = every 2–3 years, 1 point = (almost) every year)	458	1.14	1.1	236	0.80	0.9	222	1.49	1.2

*Mean = arithmetic mean; SD = standard deviation

### Data collection

The face-to-face and online surveys were conducted in parallel between June and August 2020. The same survey, created in LimeSurvey (LimeSurvey GmbH, Hamburg), was used for both samples and pre-tested in two versions (Appendix S1). The face-to-face surveys were conducted by two interviewers who read out the questions from a tablet PC and recorded the answers on the device. The survey began with a plant species identification task. Respondents were shown four photographic compilations, each comprising three photos of one plant species: one photo of the plant individual or population in its typical habitat and two more, from different distances and angles, of the plant part that is most frequently foraged and commercialised in Austria (Schunko and Vogl 2018, 2020). Since we had two sets of plant species, data were collected for eight plant species in total (Table 1). We selected plant species that are frequently foraged in Austria (Schunko and Vogl 2018, 2020), taking care to balance our selection between different life forms (herbs, shrubs, trees) and different foraged plant parts (flowers, fruits, leaves). For those plant species that the respondents correctly identified, we asked in two further questions how often they foraged those particular plant species outside of private gardens in Vienna and in the countryside. The foraging frequencies were recorded on a 4-point Likert scale (Table 1). We then asked respondents to rate eleven statements about issues that support or provide barriers to urban wild food foraging on a 5-point Likert scale. The statements included perceptions, knowledge and behaviour regarding urban wild food foraging, and were based on prior semi-structured interviews with wild food foragers in Vienna. Six of these statements were positively formulated, five negatively. Finally, we asked the respondents to rate a validated 6-item nature relatedness scale (Nisbet and Zelenski 2013) and to give socio-demographic, geographic and experience-related information on eight further variables that earlier studies have suggested are useful to explain urban foraging (Table 1). Each survey lasted between 12 and 30 min.

### Data analysis

First the variables were analysed descriptively using means, standard deviations and percentages, and then an urban and rural foraging frequency score was calculated for each respondent. To do this, we summed the foraging frequencies given for the individual plant species (0 points = never, 0.33 points = less frequently than every 2–3 years, 0.66 points = every 2–3 years, 1 point = (almost) every year). In addition, we calculated the mean of the 6-item nature relatedness scale to obtain the nature relatedness score. We then calculated Mann–Whitney tests to explore differences in foraging frequencies in urban and rural spaces and between the face-to-face and online sub-samples. Subsequently, we used the foraging frequency scores as outcome variables in two multiple linear regressions using the Enter method. The eight socio-demographic, geographic and experience-related variables in addition to the nature relatedness score were selected as predictor variables (Table 1), and the sub-sample membership and set number added to the model to control for biases. To assess foraging behaviour in greater detail, we also performed a hierarchical cluster analysis, classifying urban foragers according to their urban and rural foraging frequencies, using Ward’s method. Kruskal–Wallis and Chi^2^ tests served to characterise the clusters of foragers identified using the same eight variables already used as predictors in the multiple linear regressions. An exploratory factor analysis was performed to identify the latent constructs behind the eleven statements that could support or provide barriers to urban wild food foraging. We used principal component analysis as a factor extraction method and Varimax rotation to increase the interpretability of factors. The Kaiser–Meyer–Olkin measure (0.623) and a highly significant Bartlett’s test of sphericity (*p* < 0.001) indicated the suitability of the data for factor analysis. The Scree test and an evaluation of the resulting factors were used to select a model with three extracted factors. Factor score estimations were calculated using the regression method. Finally, we used these factor score estimations to test how the extracted factors are associated with the clusters of foragers using Kruskal–Wallis and Bonferroni post-hoc tests. The statistical analysis was performed in SPSS 24 (IBM, Armonk, New York).

## Results

### Wild food species foraged

Six of the eight wild food species depicted on the photographic compilations were identified correctly by at least 88% of respondents. Only *Tilia* sp. and *Prunus domestica subsp. insititia* (L.) Bonnier & Layens were correctly identified by a smaller percentage (68% and 62% respectively) (Table 2). Mean foraging frequencies for the eight wild food species on the 4-step Likert scale ranged from 1.4 to 2.3 (*M* = 1.9) for urban foraging and from 1.3 to 2.6 (*M* = 2.1) for rural foraging, indicating that the respondents reported that they foraged each plant species less frequently than every 2–3 years in urban and rural spaces. 71% of respondents reported that they had previously foraged for at least one of the plant species in Vienna. Respondents reported that they had foraged for *Allium ursinum* L. significantly more often in Vienna than in the countryside (*p*_MW-test_ < 0.001), for *Fragaria* sp. (*p*_MW-test_ < 0.001), *Urtica* sp. (*p*_MW-test_ = 0.017) and *Rubus fruticosus* agg. (*p*_MW-test_ = 0.001) significantly more often in the countryside than in Vienna, and for *Tilia* sp. (*p*_MW-test_ = 0.877), *Prunus domestica subsp. insititia* (L.) Bonnier & Layens (*p*_MW-test_ = 0.560), *Sambucus nigra* L. (*p*_MW-test_ = 0.210) and *Juglans regia* L. (*p*_MW-test_ = 0.143) to the same extent in Vienna and in the countryside. Dividing the plant species into two groups according to their life forms and growth height revealed that respondents reported that they foraged for *herbs-small shrubs* more frequently in the countryside than in Vienna (*p*_MW-test_ < 0.001), whereas *large shrubs-trees* were foraged to similar extents in Vienna and the countryside (*p*_MW-test_ = 0.218).

**Table 2:** Plant identification competence and foraging frequencies for eight wild food species (*n* = 458)

	Wild food species	Life form	Popular plant part^a^	Identified^b^	Foraging frequency Vienna^c^		Foraging frequency countryside^c^		Foraged in Vienna or countryside^d^	Foraged in Vienna^d^	Foraged in countryside^d^
				%	M*	SD*	*M*	SD	%	%	%
Set 1 (*n* = 239)	*Allium ursinum* L.	Herb	Leaves	92.5	2.3	1.3	1.9	1.2	66.1	55.2	38.9
	*Fragaria* sp.	Herb	Fruits	97.1	1.6	1.1	2.6	1.3	74.1	27.6	70.3
	*Tilia* sp.	Tree	Flowers	67.8	1.4	0.9	1.3	0.8	25.5	18.0	18.4
	*Prunus domestica* subsp. *insititia* (L.) Bonnier & Layens	Shrub-tree	Fruits	61.9	1.8	1.2	1.8	1.1	46.0	36.4	35.1
*M*_Set1_				79.8	1.8	1.1	1.9	1.1	52.9	34.3	40.7
Set 2 (*n* = 219)	*Urtica* sp.	Herb	Leaves	95.4	1.7	1.1	1.9	1.2	47.9	28.3	40.2
	*Rubus fruticosus* agg	Small shrub	Fruits	97.7	2.1	1.3	2.5	1.3	72.1	48.9	63.0
	*Sambucus nigra* L.	Shrub-tree	Flowers	95.9	2.1	1.3	2.2	1.3	63.5	46.6	52.5
	*Juglans regia* L.	Tree	Fruits	88.6	1.8	1.2	2.0	1.2	56.6	37.0	44.3
*M*_Set2_				94.4	1.9	1.2	2.1	1.3	60.0	40.2	50.0
*M*_Set1&2_				87.1	1.9	1.2	2.0	1.2	56.5	37.3	45.3

*M = arithmetic mean; SD = standard deviation

^a^Most popular plant part foraged in Austria as reported in Schunko and Vogl (2018, 2020)

^b^Percentage of respondents who correctly identified wild food species on photographic compilations

^c^Foraging frequency outside of private gardens measured on Likert scale ranging from 1 = never, 2 = less frequently than every 2–3 years, 3 = every 2–3 years, 4 = (almost) every year

^d^Percentage of respondents answering 2–4 on Likert scale for foraging frequencies

The online sub-sample of respondents reported that they foraged for all wild food species more frequently than those interviewed face-to-face (*p*_MW-test_ < 0.001 for all eight species). Considering only the face-to-face sub-sample of visitors of urban green spaces, 64% reported that they had previously foraged for one of the depicted plant species in Vienna.

### Characteristics of urban foragers

The regression models explained the frequency of foraging in rural areas (*R*^2^_adjusted_ = 30.7%) better than in urban areas (*R*^2^_adjusted_ = 22.9%) (Table S1). Multicollinearity was not an obstacle because the variance inflation factors (VIF) did not exceed 3.3. Two predictor variables were significantly related to both outcome variables: respondents with higher nature relatedness (*p*_urban_ < 0.001; *p*_rural_ < 0.001) and respondents having foraged at least once per year in childhood (*p*_urban_ = 0.028; *p*_rural_ < 0.001) foraged more frequently in urban and rural spaces. Nature relatedness was the strongest predictor of all the selected variables (*β*_urban_ = 0.282; *β*_rural_ = 0.250). Urban foraging frequency was lower for urban dwellers residing in the central districts of the city than for those living in peripheral districts (*p* = 0.028), whereas there was no such difference with regard to rural foraging frequency (*p* = 0.783). Respondents foraging more frequently in rural areas were significantly more often women (*p* = 0.015), younger (*p* = 0.031), had access to private gardens more often (*p* = 0.007) and had grown up in cities less often (*p* = 0.037). Duration of residence in Vienna (*p*_urban_ = 0.383; *p*_rural_ = 0.190) and higher education (*p*_urban_ = 0.597; *p*_rural_ = 0.181) did not predict any of the outcome variables. Sub-sample membership was, as expected, a significant confounding variable in both regressions (*p* < 0.001 for both). In addition, respondents reported that they foraged for plant set 2 significantly more often in rural areas than for plant set 1 (*p* = 0.014).

Hierarchical cluster analysis grouped the foragers into four clusters (Table 3). The Kruskal–Wallis and Chi^2^ tests indicated that urban and rural foraging frequencies and seven predictor variables were significantly associated with the four clusters, whereas higher education and residence in central districts were unrelated. The largest cluster (*n* = 224) comprised *occasional foragers* who had the lowest foraging frequencies in both urban (*M* = 0.4) and rural areas (*M* = 0.5). The second cluster *rural foragers* (*n* = 116), included respondents with a relatively low urban foraging frequency (*M* = 1.0), but a relatively high rural foraging frequency (*M* = 2.2), whereas the third cluster *urban foragers* (*n* = 69) showed the opposing trend. The smallest cluster *universal foragers* (*n* = 49) included those respondents with the highest urban (*M* = 3.2) and highest rural (*M* = 3.5) foraging frequencies.

**Table 3:** Hierarchical cluster analysis of urban dwellers based on their urban and rural foraging frequencies (n = 458)

Variables	Cluster 1	Cluster 2	Cluster 3	Cluster 4	*p* value
	Occasional foragers	Rural foragers	Urban foragers	Universal foragers	
Urban foraging frequency (mean)	0.4	1.0	2.4	3.2	< 0.001**^a^
Rural foraging frequency (mean)	0.5	2.2	1.2	3.5	< 0.001**^a^
Sex female (%)	61	78	76	78	0.002**^b^
Age (mean)	41.7	37.0	45.9	41.7	0.002**^a^
Higher education (mean)	1.54	1.67	1.62	1.63	0.084^a^
Residence time in Vienna (mean)	26.4	21.1	28.3	28.5	< 0.014*^a^
Residence in central districts (mean)	1.41	1.41	1.28	1.27	0.055^a^
Access to a private garden (%)	38	55	48	71	< 0.001**^b^
Childhood in a city (%)	67	48	59	63	0.010*^b^
Childhood foraging (%)					
< 1 time/year	33	23	22	18	0.052^b^
1–3 times/year	33	29	33	27	0.760^b^
> 3 times/year	34	47	45	55	0.012*^b^
Nature relatedness (mean)	3.8	4.0	4.3	4.3	< 0.001*^a^
n	224	116	69	49	
n_face-to-face survey_	155	43	31	7	
n_online survey_	69	73	38	42	

*p < 0.05. **p < 0.01

^a^Kruskal–Wallis test

^b^Chi^2^-test

### Barriers to urban foraging

The eleven statements about issues that support or provide barriers to urban foraging received agreement ratings ranging from 2.8 to 4.5 on a 5-point Likert scale (Table 4). Using exploratory factor analysis, the eleven statements were grouped into three factors, explaining 42% of the variance in the model. The first factor was composed of four statements about access to and the availability of wild foods and urban green spaces suitable for urban foraging. We chose the label *access to wild foods* to describe this factor. The second factor encompassed statements about the customary and legal knowledge and practices of foragers, and thus measured the extent to which urban foraging is appreciated and accepted by respondents. We labelled this factor *social acceptance*. The third factor compiled four statements about appropriate quantities of foraged plant materials and potential damage to plant populations. We labelled this factor *ecological impacts*. The statements grouped in the factor *ecological impacts* obtained the highest agreement (*M* = 4.3), followed by the factor *access to wild foods* (*M* = 3.2) and *social acceptance* (*M* = 3.0). The statements of the factors *access to wild foods* and *social acceptance* asked about respondents’ perceptions, while the statements of the factor *ecological impacts* asked about respondents’ knowledge and behaviour. Given that the more respondents agreed with a factor, the more favourable it was for urban foraging, their knowledge about ecological impacts and behaviour were of less concern, while access to green spaces and social acceptance were more often perceived as barriers to foraging in the city.

**Table 4:** Exploratory factor analysis of statements on issues that support or put up barriers to urban foraging (*n* = 407)

Statements	Mean**^a^	SD**	Factors^b^		
			Access to wild foods	Social acceptance	Ecological impacts
There are many easily accessible green spaces in Vienna for wild food foraging*	3.3	1.3	0.735		
There are many opportunities for wild food foraging in Vienna	3.5	1.2	0.697		
Hardly any of the green spaces in Vienna are too contaminated or polluted to forage for wild food*	3.2	1.2	0.695		
The authorities responsible for the green spaces in Vienna are not sceptical about wild food foraging*	2.9	1.0	0.522		
Foragers are careful when foraging for wild food in Vienna	3.3	1.1		0.714	
Foragers in Vienna know where they are allowed to forage for wild food	2.8	1.3		0.680	
The Viennese like to see wild food being foraged in Vienna	2.9	1.1		0.566	
I deliberately leave some plants behind when I forage	4.5	0.9			0.629
I almost never forage for more than I need*	4.3	1.1			0.594
In high meadows it is wrong to forage in the middle of the meadows*	3.9	1.1			0.442
When harvesting from trees it is not usual for branches to break*	4.4	1.0			0.426

*Statements were formulated in opposite directions in the survey

**Mean = arithmetic mean; SD = standard deviation;

^a^response options on a 5-step Likert scale ranging from 1 = full disagreement to 5 = full agreement

^b^factor loadings below 0.4 are suppressed

Finally, we tested the independence of the three factors from the four clusters of foragers (Table S2). *Access to wild foods* and *ecological impacts* were significantly related to clusters of foragers (*p* < 0.001 for both), whereas the perception of *social acceptance* was not (*p* = 0.780). *Urban foragers* rated the factor *access to wild foods* significantly more highly than *rural foragers* (*p* < 0.001), and thus perceived access to be less problematic. *Urban foragers* and *universal foragers* had significantly higher factor scores relating to *ecological impacts* compared with *rural foragers* (*p* = 0.009 for *rural–urban foragers*; *p* = 0.036 for *rural-universal foragers*) and *occasional foragers* (*p* < 0.001 for both), and thus had better knowledge and behaviour to avoid the negative ecological impacts of urban foraging.

## Discussion

Four key findings were derived from this study that can inform the planning of urban environments for increased human-nature interactions through urban foraging. Before discussing them in detail, we raise a methodological concern. We identified respondents using two different sampling strategies: face-to-face and online. In this way we were able to survey respondents along the entire spectrum from non-foragers to intensive foragers. Neither face-to-face surveys nor online surveys alone would have provided access to this spectrum. Indeed, with face-to-face surveys we hardly reached intensive foragers, while with online surveys we hardly reached the inexperienced foragers. However, the disadvantage of splitting our sampling strategy was that we ended up with a small sample size, especially when the sub-samples are analysed individually. This is relevant because our first key finding below is based only on the subsample of face-to-face surveys. This result should, therefore, be treated with caution. The other three key findings are based on the analysis of both sub-samples together and are, therefore, more reliable, even though the sample size of this study does not yield representative results for a city with almost 2 million inhabitants.

Our first key finding is consistent with previous studies that found that urban wild food foraging is a widely adopted practice in urban areas, and thus enables human-nature interactions for many urban dwellers. While one third of the urban dwellers interviewed in Berlin (Fischer and Kowarik 2020) and 26% in Atlanta (Gaither et al. 2020) reported that they forage, this figure was 64% of the sub-sample of visitors to urban green spaces in Vienna. This higher percentage for Vienna is probably due to different sampling strategies and likely overestimates the actual proportion of urban foragers in Vienna. While in Berlin urban dwellers were interviewed in different types of urban spaces and in Atlanta a random sample was drawn, here we interviewed visitors to urban green spaces, who tend to forage more frequently than other urban dwellers (Fischer and Kowarik 2020). Our results still reveal that a significant number of urban dwellers forage from time to time, whereas foraging frequencies were much lower compared with other cities. For example, in two medium-sized South African towns, more than half of the people who forage reported that they foraged for wild foods several times a week (Garekae and Shackleton 2020). In contrast, most foragers in Vienna gather the selected, very popular wild food species less than once a year. This is also consistent with the fact that in five European cities, hardly any urban dweller reports foraging as their main activity when visiting urban nature (Fischer et al. 2018). Considering that many urban dwellers forage from time to time, but that this activity is largely unsupported in Vienna as elsewhere (Shackleton et al. 2017), it underlines the potential of urban foraging to increase human–nature interactions and the nature relatedness of urban dwellers.

Second, we found that urban dwellers not only forage in the city but in the countryside too, and that the frequency of foraging in urban or rural spaces relates to the targeted wild food species and their life forms. The first part of this result is in line with the finding that urban dwellers make planned outings to rural spaces and forage there incidentally when opportunities arise (McLain et al. 2013; Landor-Yamagata et al. 2018). What is new, to the best of our knowledge, is that herbaceous plant species and small shrubs are foraged more frequently in the countryside than in the city, whereas there is no such difference when it comes to large shrubs and trees. This pattern in foraging might be linked to the availability and accessibility of wild food species in the urban space. For example, *Tilia* sp. is one of the most commonly planted trees in streets and parks in Vienna (MA42 2021), and the other large shrubs included in this study, *Prunus domestica subsp. insititia* (L.) Bonnier & Layens and *Sambucus nigra* L., also grow in parks across the city. In comparison, herbaceous wild food species and thorny shrubs, such as *Urtica* sp. and *Rubus fruticosus* agg., tend to be mown and removed from managed public urban green spaces. In addition, low-growing wild food species are more affected by contamination by dogs, a major problem for urban foraging (Fischer and Kowarik 2020), than large shrubs and trees. Finding herbaceous wild food species and smaller shrubs of good quality is therefore difficult in more frequented public urban green spaces, while large shrubs and trees are accessible there (Landor-Yamagata et al. 2018). One exception is *Allium ursinum* L., which is foraged more frequently in Vienna than in the countryside, although it is herbaceous. This is because *A. ursinum* grows in large populations in forests in and on the edge of Vienna, and is the most frequently known and foraged wild food species in Austria (Schunko and Vogl 2020). The plant species, therefore, has high cultural value and is widely available for foragers in the city, and contaminated sites can be easily avoided. This shows how much foraging depends on the availability of good-quality plant material. Suggestions to support greater availability include planting edible trees and shrubs, enhancing vegetation diversity, allowing more tolerance towards spontaneous vegetation or creating dog-free sites where foraging is permitted (Palliwoda et al. 2017; Fischer and Kowarik 2020). Our results show that in addition to these suggestions, particular attention should be paid to herbaceous wild food species if foraging in the city is to be promoted.

Third, urban foragers in Vienna share the characteristics of pronounced nature relatedness, childhood foraging experiences and residence on the periphery of the city, but they are heterogeneous in terms of their sex, age, education and length of residence in the city. These findings overlap with the findings that the socio-demographic backgrounds of urban dwellers are diverse (Robbins et al. 2008; Gianotti and Hurley 2016; Fischer and Kowarik 2020; Garekae and Shackleton 2020), that childhood foraging experience is a suitable predictor of foraging (Garekae and Shackleton 2020) and that the degree of urbanisation of the place of residence is positively associated with foraging (Schlesinger et al. 2015; Gianotti and Hurley 2016; Garekae and Shackleton 2020; Somesh et al. 2021). In addition to the last finding, this study showed that residents of highly urbanised districts forage in the countryside to the same extent as residents of peripheral districts. This indicates that the residents of highly urbanised districts are generally not less inclined to forage, but that lack of access to public urban green spaces in their neighbourhoods may be the main reason why they forage less in the city. This again highlights the relevance of the availability of wild foods nearby for encouraging urban foraging.

Nature relatedness has previously been suggested to be associated with urban foraging (McLain et al. 2013; Fischer and Kowarik 2020) but, to the best of our knowledge, this has not been empirically tested. What has been tested however, although found to be unrelated to foraging, is nature contact (Fischer and Kowarik 2020). The concept of nature contact is narrower than the concept of nature relatedness, however, which includes the emotional, spiritual and knowledge-based connectedness with nature in addition to awareness of and contact with nature (Nisbet and Zelenski 2013). The extent of contact with nature and time spent in nature alone is, therefore, not a predictor of urban foraging, while urban dwellers who also feel emotionally and spiritually connected with nature are more likely to engage in urban foraging. Taking into account that nature relatedness is enhanced by meaningful and emotional contact with nature (Lumber et al. 2017) and psychological proximity to nature through touching or smelling (Colléony et al. 2020), and that these are core aspects of foraging (Poe et al. 2014), urban foraging might not only relate to relatedness with nature but even reinforce it. In view of the fact that nature relatedness enhances human health and wellbeing (Martin et al. 2020; Myers 2020), these results confirm that urban foraging could be a promising strategy for improving the health and wellbeing of urban dwellers.

Fourth, our results suggest that negative ecological impacts are less of an issue, but that social acceptance and lack of access to wild foods present barriers to urban foraging. Most foragers in Vienna knew how to avoid the negative ecological impacts of foraging and reported that they were careful. Although the four questions in the survey relating to negative ecological impacts did not cover all facets of the topic, the consistently high percentage of favourable answers indicated that knowledge of foraging to minimise negative ecological impacts was widespread. This result is a cause for optimism about the potential of foraging in urban areas, and is in contrast to the scepticism public authorities tend to have (Poe et al. 2013; Hurley et al. 2015; Shackleton et al. 2017). In addition, our study showed that urban dwellers who forage a lot in urban spaces tend to be more knowledgeable about ecological impacts than urban dwellers who mainly forage in rural spaces. Thus, urban foragers seem to be aware of the need to be particularly careful with urban vegetation, which can be easily damaged due to the potentially large numbers of foragers and the limited amount of public urban green spaces that can be used for foraging. However, even if knowledge and practices preventing and minimising negative ecological impacts are widespread, this does not mean that there are no ecological impacts of foraging, because even a small number of inexperienced or careless foragers can quickly cause considerable damage to urban vegetation, for example if foraging for rare species or breaking branches (Schunko et al. 2021). However, our results confirmed that targeted measures to identify and prevent such damaging practices are needed to protect urban vegetation rather than a general restriction of access to wild foods for urban dwellers.

Although we found that urban foragers have good levels of knowledge for limiting negative ecological impacts and they reported practices of care, there was only moderate social acceptance. This is in line with other studies that found that urban dwellers neither strongly support nor strongly object to foraging (Gianotti and Hurley 2016). However, this moderate level of social acceptance can present barriers for tentative foragers and discredit urban foraging, even though it is a legitimate activity. Improving public opinions about foraging would thus be valuable for supporting the practice. This could be achieved by informing urban dwellers about the legitimacy of the activity and highlighting the manifold potentials of foraging for urban dwellers and urban green spaces, including tangible improvements to urban green spaces by their stewardship practices (McLain et al. 2013, 2017). The barrier of lack of access to wild foods was perceived differently by different urban dwellers. In particular, respondents who mainly foraged in rural areas reported a lack of access for foraging in urban areas, while this was less of an issue for respondents who mainly foraged in urban areas. This suggests that better information on the legal framework for foraging, availability of wild foods and safety of urban wild foods could improve access to wild foods for all urban dwellers. These barriers to foraging are not unique to Vienna. Legal frameworks commonly restrict access for urban foragers (McLain et al. 2013; Shackleton et al. 2017), and the contamination of urban green spaces (Mollee et al. 2017; Fischer and Kowarik 2020) and lack of availability of wild foods (Garekae and Shackleton 2020; Somesh et al. 2021) are concerns that have been raised by urban dwellers in other cities too. Barriers to urban foraging thus appear to be similar across geographical and social contexts.

## Conclusions

With this study, we add to the growing body of literature finding that urban foraging is widespread in cities around the globe, whereas barriers inhibit its true potential. To reduce these barriers, public authorities should recognise urban foraging as an opportunity to enable urban dwellers' interaction with nature, health and well-being. The barriers then need to be addressed at three levels: the legal frameworks that regulate uses of public urban green spaces need to acknowledge urban foraging as legitimate activity; the planning and management of public urban green spaces needs to make sure that green spaces and plant species suitable for foraging are accessible for urban dwellers in many parts of urban areas, not only in peripheral districts; and urban dwellers need to be actively informed about foraging regulations and food safety of foraged plant parts. These levels of barriers appear to be similar for cities around the globe. However, to address them, attention needs to be paid to local conditions, such as ownership and access arrangements to public urban green spaces, or plant species and plant life forms favoured by local foragers. The involvement of foragers in planning processes is therefore essential in order to develop adequate, locally adapted solutions.

## Supplementary Information

Below is the link to the electronic supplementary material.

Supplementary file1 (PDF 5079 kb)
