# Supplementary material for: Urban nature at the fingertips: Investigating wild food foraging to enable nature interactions of urban dwellers
Source: Ambio. 2021 Oct 28;51(5):1168–78. doi: 10.1007/s13280-021-01648-1 (PMC8552430; doi:10.1007/s13280-021-01648-1)
Supplement: Supplementary file 1 — Supplementary file1 (PDF 5079 kb) [file 13280_2021_1648_MOESM1_ESM.pdf]

***Ambio***

Supplementary Information
*This supplementary information has not been peer reviewed.*

Title: Urban nature at our fingertips: Investigating wild food foraging to enhance the nature relatedness of urban dwellers

Authors: Christoph Schunko, Anjoulie Brandner

**Table S1: Multiple linear regression models predicting urban and rural foraging frequency (n=436)**

| | Urban foraging frequency | | | Rural foraging frequency | | | VIF |
|---|---|---|---|---|---|---|---|
| Predictors | β | t | p-value | β | t | p-value | |
| (Constant) | | -5.140 | 0.000 | | -2.279 | 0.023 | |
| Age | 0.092 | 1.299 | 0.195 | -0.146 | -2.159 | 0.031* | 2.855 |
| Sex | -0.026 | -0.592 | 0.554 | -0.103 | -2.446 | 0.015* | 1.104 |
| Higher education | -0.024 | -0.529 | 0.597 | 0.058 | 1.340 | 0.181 | 1.180 |
| Residence duration Vienna | 0.066 | 0.873 | 0.383 | 0.094 | 1.312 | 0.190 | 3.233 |
| Residence central districts | 0.095 | 2.210 | 0.028* | -0.011 | -0.276 | 0.783 | 1.050 |
| Access private garden | 0.058 | 1.311 | 0.190 | 0.112 | 2.692 | 0.007** | 1.089 |
| Childhood in city | -0.013 | -0.273 | 0.785 | -0.094 | -2.089 | 0.037* | 1.271 |
| Childhood foraging <1 times/year | -0.106 | -2.202 | 0.028* | -0.172 | -3.762 | 0.000** | 1.311 |
| Childhood foraging 1-3 times/year | -0.030 | -0.627 | 0.531 | -0.102 | -2.239 | 0.026* | 1.299 |
| Nature relatedness | 0.282 | 6.341 | 0.000** | 0.250 | 5.913 | 0.000** | 1.119 |
| Set number | 0.039 | 0.907 | 0.365 | 0.100 | 2.478 | 0.014* | 1.023 |
| Sub-sample | 0.364 | 7.536 | 0.000** | 0.371 | 8.087 | 0.000** | 1.318 |
| $R^2_{adjusted}$ | | 0.229 | | | 0.307 | | |

*p<0.05. **p<0.01

**Table S2: Differences between clusters of foragers in their perception of barriers for urban foraging (n=407)**

| Clusters | Factors | | |
|---|---|---|---|
| | Access to wild foods | Social acceptance | Ecological impact |
| | $p_{KW\text{-}test}$ [1] | $p_{KW\text{-}test}$ [1] | $p_{KW\text{-}test}$ [1] |
| Clusters of urban foraging frequency | <0.001** | 0.780 | <0.001** |
| Pairwise comparisons of clusters | $p_{BF\text{-}PostHoc\text{-}test}$ [2] | $p_{BF\text{-}PostHoc\text{-}test}$ [2] | $p_{BF\text{-}PostHoc\text{-}test}$ [2] |
| Occasional – Rural foragers | 0.107 | na | 0.353 |
| Rural – Universal foragers | 0.058 | na | 0.036* |
| Rural – Urban foragers | <0.001** | na | 0.009** |
| Occasional – Universal foragers | 1.000 | na | <0.001** |
| Occasional – Urban foragers | 0.091 | na | <0.001** |
| Urban – Universal foragers | 1.000 | na | 1.000 |

*p<0.05. **p<0.01

[1]Kruskal-Wallis test

[2]Bonferroni post hoc test

**Appendix S1: Survey questionnaire**

**Photographic compilations shown in survey questions A1 to H1**

A1 - *Allium ursinum* L.

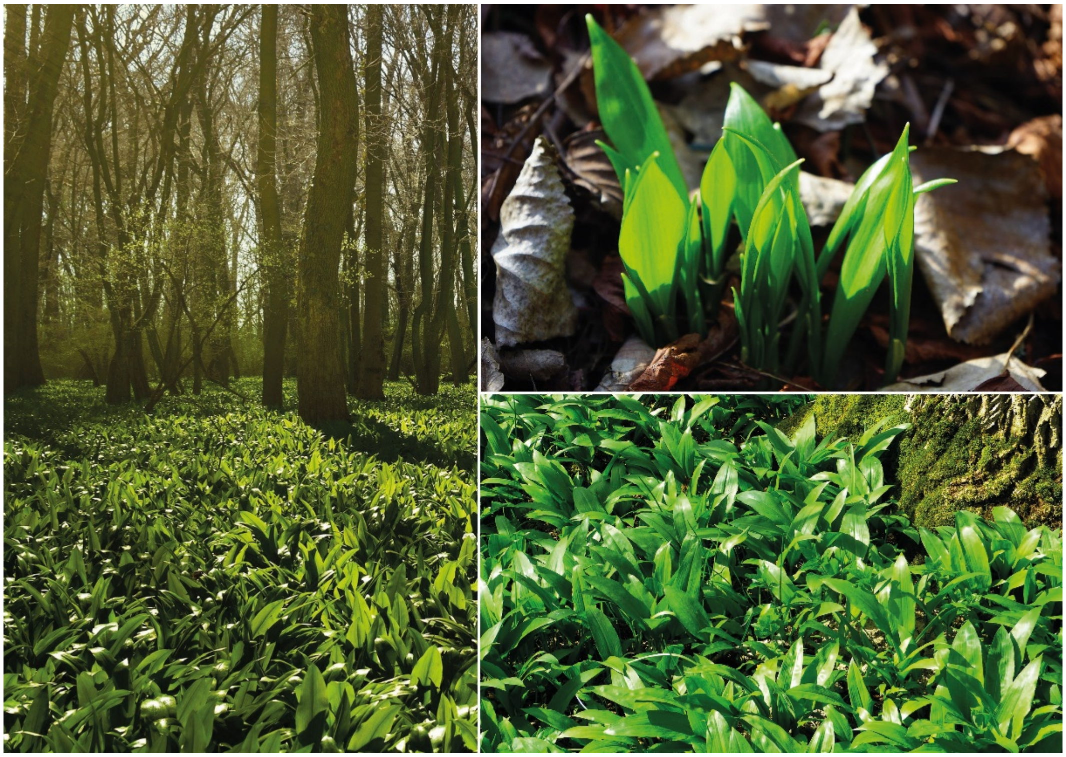

B1 - *Fragaria* sp.

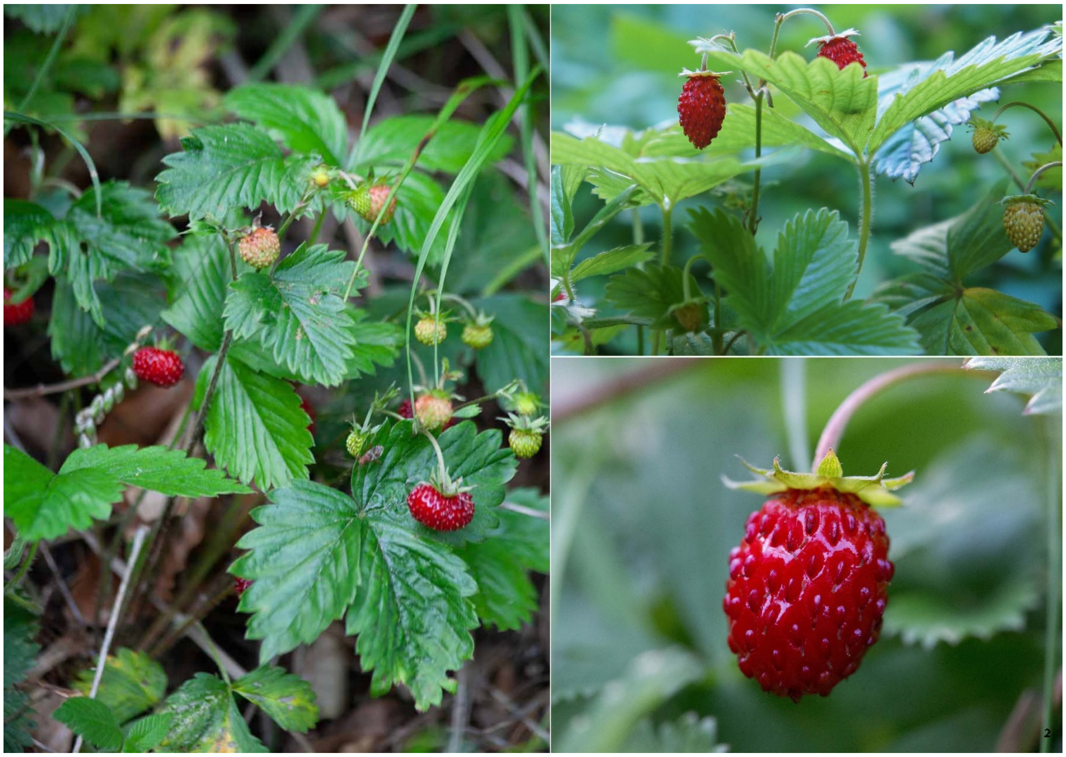

C1 - *Tilia* sp.

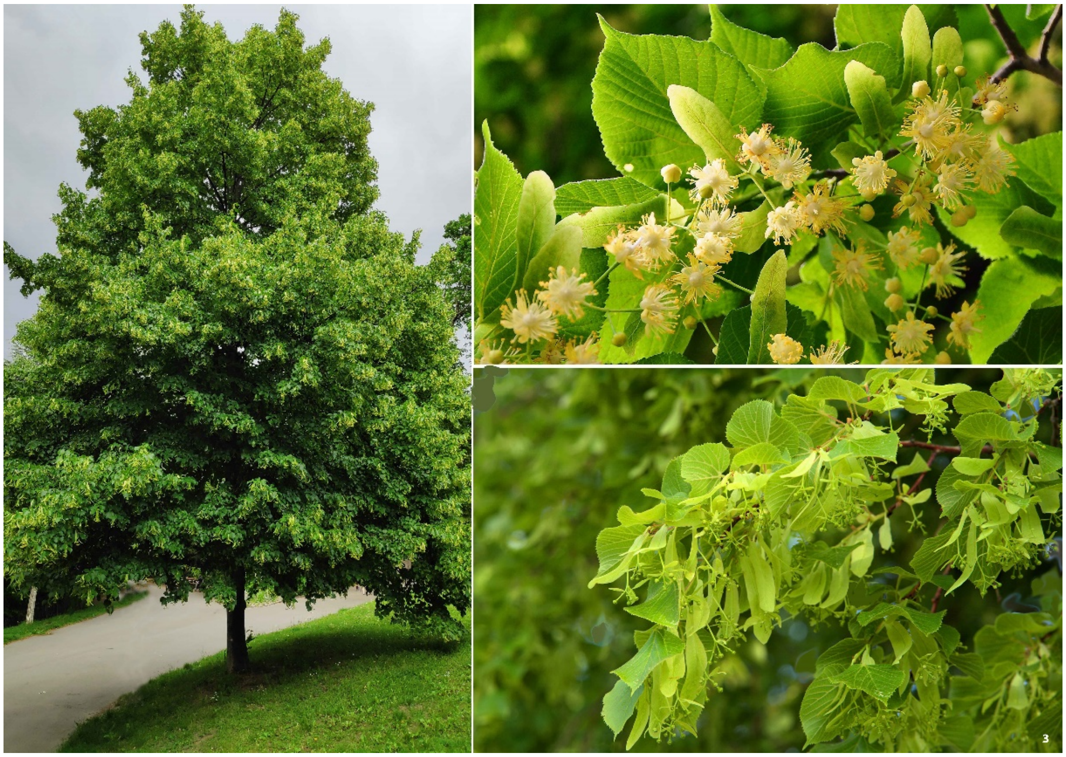


D1 - *Prunus domestica* subsp. *insititia* (L.) Bonnier & Layens

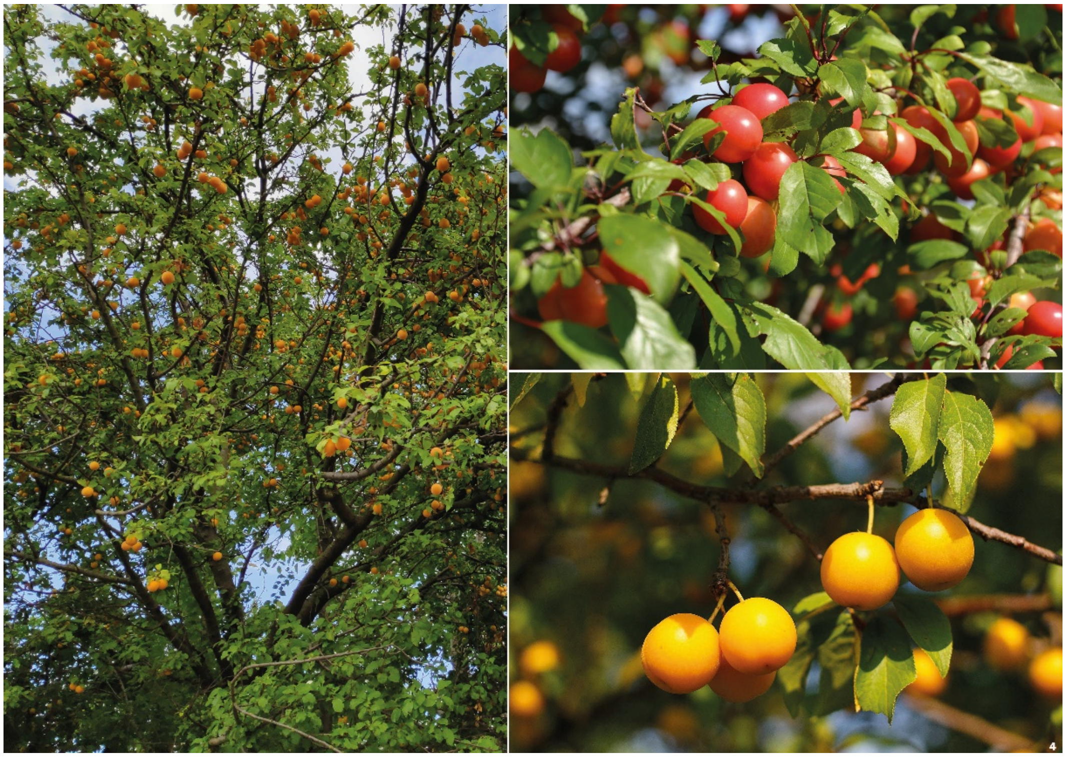

E1 - *Urtica* sp.

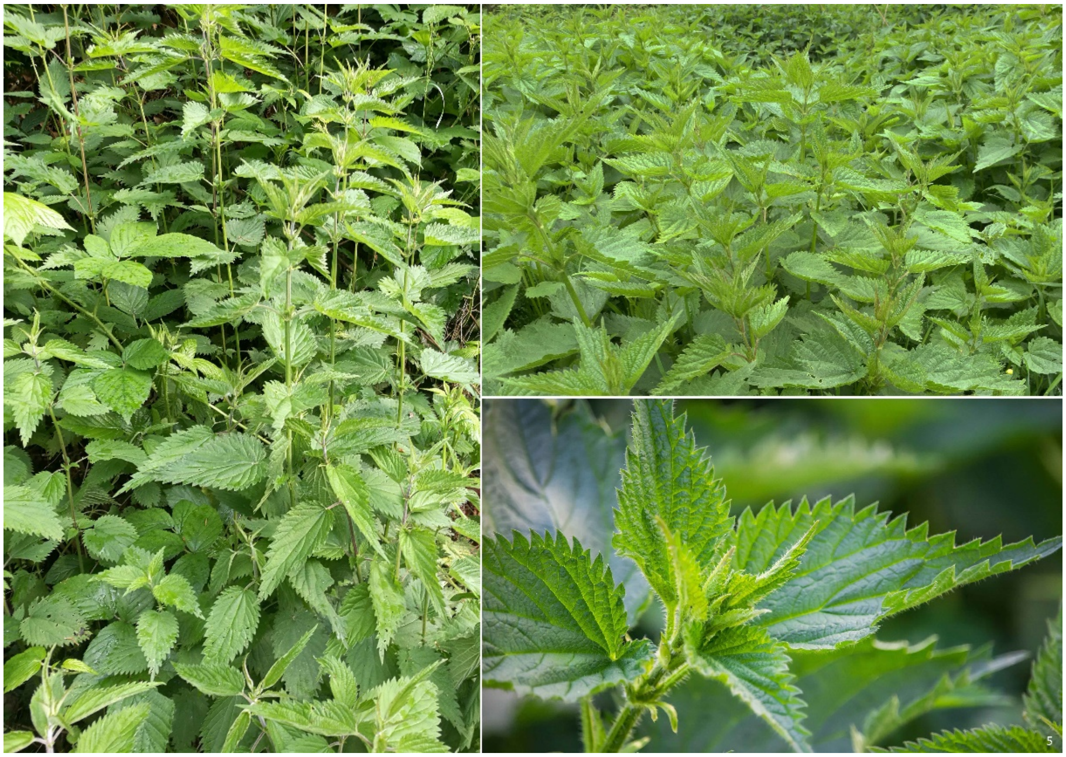


F1 - *Rubus fruticosus* agg.

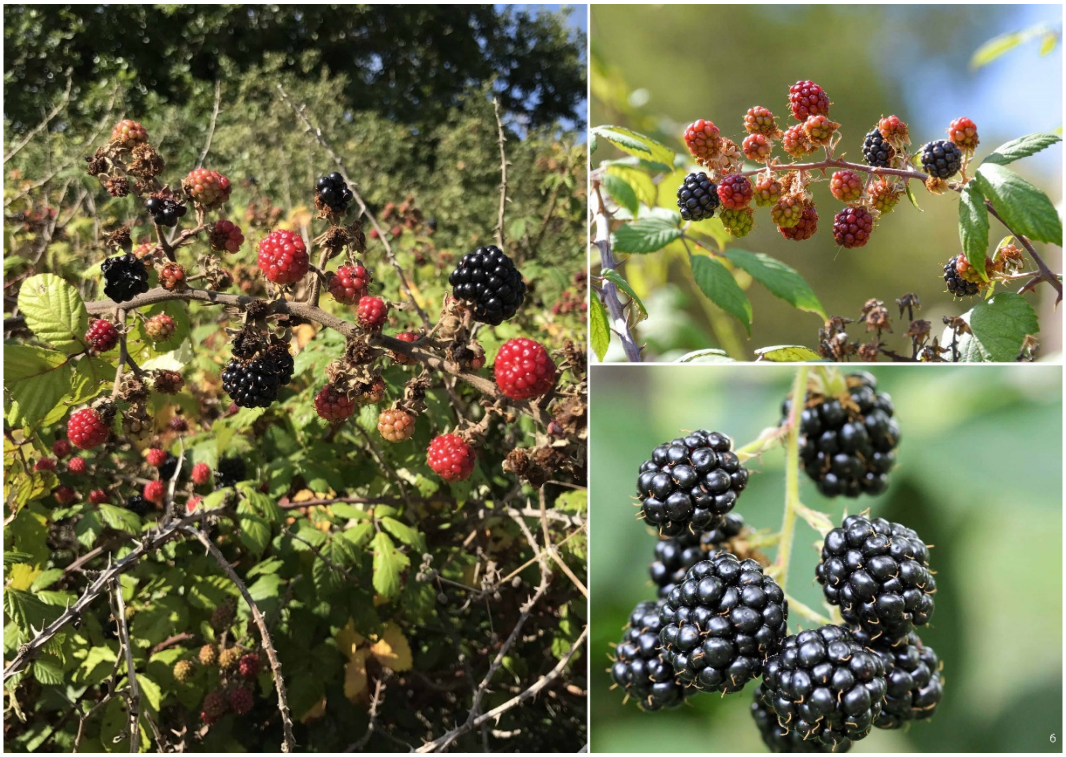

G1 - *Sambucus nigra* L.

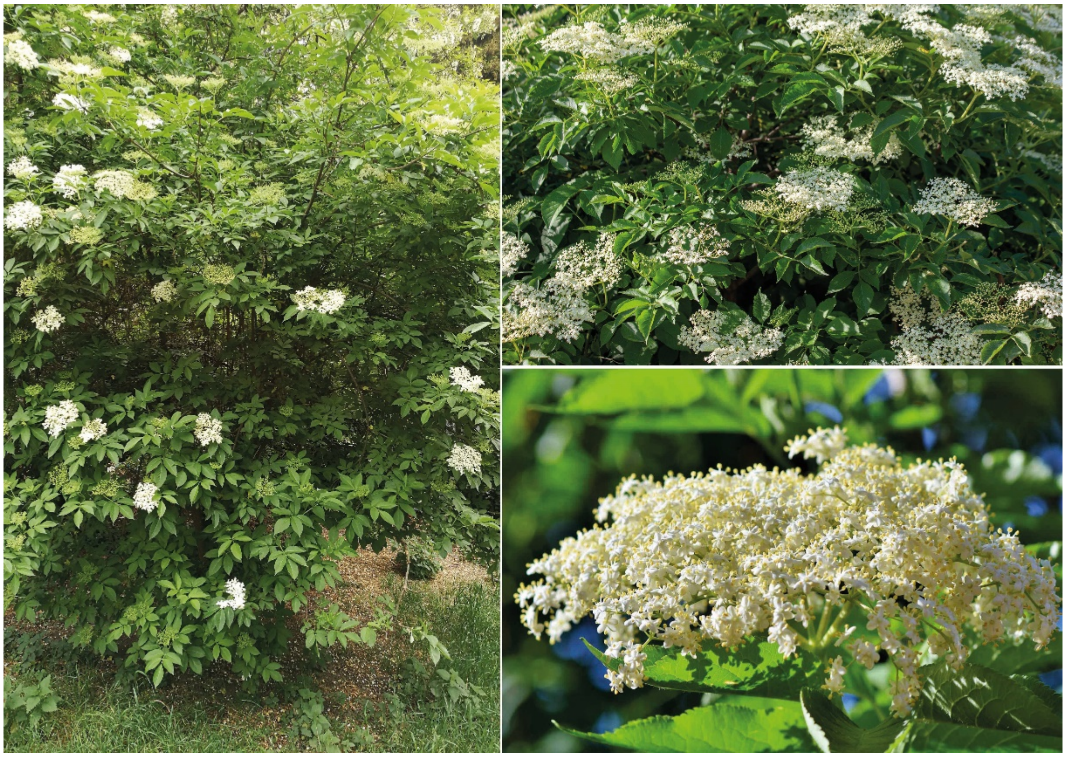

H1 - *Juglans regia* L.

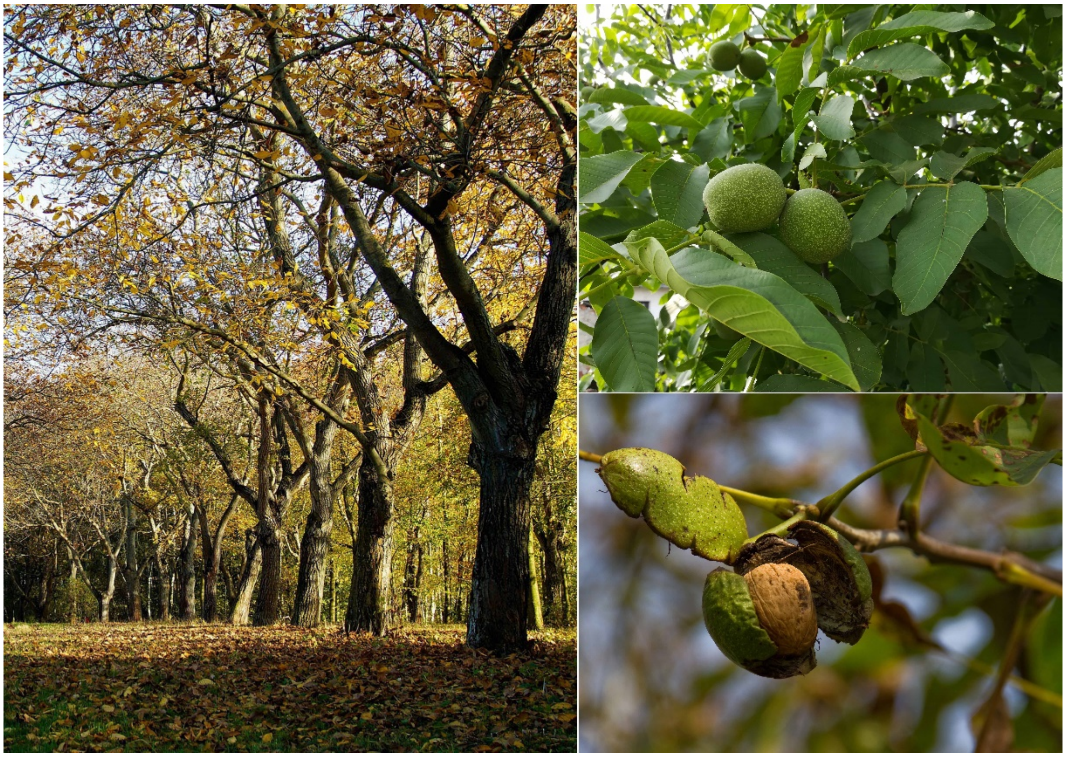

Zum Einstieg ein paar Fragen zu spezifischen Wildpflanzenarten.

**A1. Kennen Sie diese Pflanze?**

- Ja ☐
- Nein ☐

**A2. Wie nennen Sie diese Pflanze?**

**A3. Haben Sie diese Pflanze in den letzten 5 Jahren in Wien außerhalb von privaten Gärten gesammelt?**

- Ja, (fast) jedes Jahr. ☐
- Ja, alle 2-3 Jahre. ☐
- Ja, seltener als alle 3 Jahre. ☐
- Nein, nie. ☐

**A4. Haben Sie diese Pflanze in den letzten 5 Jahren am Land außerhalb von privaten Gärten gesammelt?**

- Ja, (fast) jedes Jahr. ☐
- Ja, alle 2-3 Jahre. ☐
- Ja, seltener als alle 3 Jahre. ☐
- Nein, nie. ☐

Zum Einstieg ein paar Fragen zu spezifischen Wildpflanzenarten.

**B1. Kennen Sie diese Pflanze?**

- Ja ☐
- Nein ☐

**B2. Wie nennen Sie diese Pflanze?**

**B3. Haben Sie diese Pflanze in den letzten 5 Jahren in Wien außerhalb von privaten Gärten gesammelt?**

- Ja, (fast) jedes Jahr. ☐
- Ja, alle 2-3 Jahre. ☐
- Ja, seltener als alle 3 Jahre. ☐
- Nein, nie. ☐

**B4. Haben Sie diese Pflanze in den letzten 5 Jahren am Land außerhalb von privaten Gärten gesammelt?**

- Ja, (fast) jedes Jahr. ☐
- Ja, alle 2-3 Jahre. ☐
- Ja, seltener als alle 3 Jahre. ☐
- Nein, nie. ☐

Zum Einstieg ein paar Fragen zu spezifischen Wildpflanzenarten.

**C1. Kennen Sie diese Pflanze?**

- Ja ☐
- Nein ☐

**C2. Wie nennen Sie diese Pflanze?**

**C3. Haben Sie diese Pflanze in den letzten 5 Jahren in Wien außerhalb von privaten Gärten gesammelt?**

- Ja, (fast) jedes Jahr. ☐
- Ja, alle 2-3 Jahre. ☐
- Ja, seltener als alle 3 Jahre. ☐
- Nein, nie. ☐

**C4.** **Haben Sie diese Pflanze in den letzten 5 Jahren am Land außerhalb von privaten Gärten gesammelt?**

- Ja, (fast) jedes Jahr. ☐
- Ja, alle 2-3 Jahre. ☐
- Ja, seltener als alle 3 Jahre. ☐
- Nein, nie. ☐

Zum Einstieg ein paar Fragen zu spezifischen Wildpflanzenarten.

**D1.** **Kennen Sie diese Pflanze?**

- Ja ☐
- Nein ☐

**D2.** **Wie nennen Sie diese Pflanze?**

**D3.** **Haben Sie diese Pflanze in den letzten 5 Jahren in Wien außerhalb von privaten Gärten gesammelt?**

- Ja, (fast) jedes Jahr. ☐
- Ja, alle 2-3 Jahre. ☐
- Ja, seltener als alle 3 Jahre. ☐
- Nein, nie. ☐

**D4.** **Haben Sie diese Pflanze in den letzten 5 Jahren am Land außerhalb von privaten Gärten gesammelt?**

- Ja, (fast) jedes Jahr. ☐
- Ja, alle 2-3 Jahre. ☐
- Ja, seltener als alle 3 Jahre. ☐
- Nein, nie. ☐

Zum Einstieg ein paar Fragen zu spezifischen Wildpflanzenarten.

**E1. Kennen Sie diese Pflanze?**

- [ ] Ja
- [ ] Nein

**E2. Wie nennen Sie diese Pflanze?**

**E3. Haben Sie diese Pflanze in den letzten 5 Jahren in Wien außerhalb von privaten Gärten gesammelt?**

- [ ] Ja, (fast) jedes Jahr.
- [ ] Ja, alle 2-3 Jahre.
- [ ] Ja, seltener als alle 3 Jahre.
- [ ] Nein, nie.

**E4. Haben Sie diese Pflanze in den letzten 5 Jahren am Land außerhalb von privaten Gärten gesammelt?**

- [ ] Ja, (fast) jedes Jahr.
- [ ] Ja, alle 2-3 Jahre.
- [ ] Ja, seltener als alle 3 Jahre.
- [ ] Nein, nie.

Zum Einstieg ein paar Fragen zu spezifischen Wildpflanzenarten.

**F1. Kennen Sie diese Pflanze?**

- [ ] Ja
- [ ] Nein

**F2. Wie nennen Sie diese Pflanze?**

**F3.** **Haben Sie diese Pflanze in den letzten 5 Jahren in Wien außerhalb von privaten Gärten gesammelt?**

- Ja, (fast) jedes Jahr. ☐
- Ja, alle 2-3 Jahre. ☐
- Ja, seltener als alle 3 Jahre. ☐
- Nein, nie. ☐

**F4.** **Haben Sie diese Pflanze in den letzten 5 Jahren am Land außerhalb von privaten Gärten gesammelt?**

- Ja, (fast) jedes Jahr. ☐
- Ja, alle 2-3 Jahre. ☐
- Ja, seltener als alle 3 Jahre. ☐
- Nein, nie. ☐

Zum Einstieg ein paar Fragen zu spezifischen Wildpflanzenarten.

**G1.** **Kennen Sie diese Pflanze?**

- Ja ☐
- Nein ☐

**G2.** **Wie nennen Sie diese Pflanze?**

**G3.** **Haben Sie diese Pflanze in den letzten 5 Jahren in Wien außerhalb von privaten Gärten gesammelt?**

- Ja, (fast) jedes Jahr. ☐
- Ja, alle 2-3 Jahre. ☐
- Ja, seltener als alle 3 Jahre. ☐
- Nein, nie. ☐

**G4.** **Haben Sie diese Pflanze in den letzten 5 Jahren am Land außerhalb von privaten Gärten gesammelt?**

Ja, (fast) jedes Jahr. ☐

Ja, alle 2-3 Jahre. ☐

Ja, seltener als alle 3 Jahre. ☐

Nein, nie. ☐

Zum Einstieg ein paar Fragen zu spezifischen Wildpflanzenarten.

**H1.** **Kennen Sie diese Pflanze?**

Ja ☐

Nein ☐

**H2.** **Wie nennen Sie diese Pflanze?**

**H3.** **Haben Sie diese Pflanze in den letzten 5 Jahren in Wien außerhalb von privaten Gärten gesammelt?**

Ja, (fast) jedes Jahr. ☐

Ja, alle 2-3 Jahre. ☐

Ja, seltener als alle 3 Jahre. ☐

Nein, nie. ☐

**H4.** **Haben Sie diese Pflanze in den letzten 5 Jahren am Land außerhalb von privaten Gärten gesammelt?**

Ja, (fast) jedes Jahr. ☐

Ja, alle 2-3 Jahre. ☐

Ja, seltener als alle 3 Jahre. ☐

Nein, nie. ☐

Jetzt einige Fragen dazu wie Sie das Sammeln von Wildpflanzen in Wien wahrnehmen.

**I1. Bitte bewerten Sie diese Aussagen danach wie sehr Sie ihnen zustimmen.**

**(1=Stimme sehr zu; 2=Stimme eher zu; 3=Stimme weder zu noch nicht zu, 4=Stimme eher nicht zu; 5=Stimme überhaupt nicht zu)**

| | 1 - Stimme sehr zu | 2 | 3 | 4 | 5 - Stimme überhaupt nicht zu |
|---|---|---|---|---|---|
| In Wien gibt es wenige gut erreichbare Grünflächen um essbare Wildpflanzen zu sammeln. | ☐ | ☐ | ☐ | ☐ | ☐ |
| In Wien gibt es viele Möglichkeiten essbare Wildpflanzen zu sammeln. | ☐ | ☐ | ☐ | ☐ | ☐ |
| Die in Wien für die Grünflächen verantwortlichen Behörden sind dem Sammeln gegenüber skeptisch. | ☐ | ☐ | ☐ | ☐ | ☐ |
| In Wien sind die meisten Grünflächen zu kontaminiert oder verschmutzt, um essbare Wildpflanzen zu sammeln. | ☐ | ☐ | ☐ | ☐ | ☐ |
| Die SammlerInnen in Wien wissen wo sie essbare Wildpflanzen sammeln dürfen und wo nicht. | ☐ | ☐ | ☐ | ☐ | ☐ |
| Die Wiener und Wienerinnen sehen es gerne wenn in Wien essbare Wildpflanzen gesammelt werden. | ☐ | ☐ | ☐ | ☐ | ☐ |
| Die SammlerInnen in Wien gehen beim Sammeln essbarer Wildpflanzen vorsichtig vor. | ☐ | ☐ | ☐ | ☐ | ☐ |

Hier einige Fragen zum Sammeln an sich.

**J1. Bitte bewerten Sie diese Aussagen danach wie sehr Sie ihnen zustimmen.**

**(1=Stimme sehr zu, 2=Stimme eher zu, 3=Stimme weder zu noch nicht zu, 4= Stimme eher nicht zu, 5=Stimme überhaupt nicht zu)**

| | 1 - Stimme sehr zu | 2 | 3 | 4 | 5 - Stimme überhaupt nicht zu |
|---|---|---|---|---|---|
| Häufig vorkommende Pflanzen, Blüten oder Früchte können über den Eigenbedarf hinausgehend gesammelt werden. | ☐ | ☐ | ☐ | ☐ | ☐ |
| Ich lasse beim Sammeln einige Pflanzen bewusst zurück. | ☐ | ☐ | ☐ | ☐ | ☐ |
| Das Sammeln hinterlässt keine auffälligen Spuren am Sammelplatz. | ☐ | ☐ | ☐ | ☐ | ☐ |
| Das Sammeln von Wurzeln schadet dem Vorkommen von Wildpflanzen. | ☐ | ☐ | ☐ | ☐ | ☐ |
| Bei hoch gewachsenen Wiesen ist es gut in der Mitte der Wiesen zu sammeln. | ☐ | ☐ | ☐ | ☐ | ☐ |
| Beim Beernten von Bäumen ist es normal wenn Äste abbrechen. | ☐ | ☐ | ☐ | ☐ | ☐ |
| Ich sammle manchmal mehr, als ich dann brauche. | ☐ | ☐ | ☐ | ☐ | ☐ |

Hier noch einige allgemeine Fragen zu Ihrer Beziehung zu Natur und Umwelt.

**K1. Bitte bewerten Sie diese Aussagen danach wie sehr Sie ihnen zustimmen.**

**(1=Stimme sehr zu, 2=Stimme eher zu, 3=Stimme weder zu noch nicht zu, 4=Stimme eher nicht zu, 5=Stimme überhaupt nicht zu)**

| | 1- Stimme sehr zu | 2 | 3 | 4 | 5 - Stimme überhaupt nicht zu |
|---|---|---|---|---|---|
| Mein idealer Urlaubsort wäre ein abgelegenes Wildnisgebiet. | ☐ | ☐ | ☐ | ☐ | ☐ |
| Ich nehme die Tierwelt zur Kenntnis wo immer ich bin. | ☐ | ☐ | ☐ | ☐ | ☐ |
| Meine Beziehung zur Natur ist ein wichtiger Teil dessen was ich bin. | ☐ | ☐ | ☐ | ☐ | ☐ |
| Ich fühle mich mit allen Lebewesen und der Erde sehr verbunden. | ☐ | ☐ | ☐ | ☐ | ☐ |
| Ich denke immer darüber nach wie sich mein Handeln auf die Umwelt auswirkt. | ☐ | ☐ | ☐ | ☐ | ☐ |
| Meine Verbindung zu Natur und Umwelt ist ein Teil meiner Spiritualität. | ☐ | ☐ | ☐ | ☐ | ☐ |

Abschließend noch ein paar Fragen zu Ihrer Person.

**L1. Haben Sie vor Ihrem 16.Lebensjahr überwiegend in einer Stadt mit über 10.000 EinwohnerInnen gewohnt?**

Ja ☐

Nein ☐

**L2. Wie oft haben Sie vor Ihrem 16.Lebensjahr in etwa essbare Wildpflanzen gesammelt?**

Seltener als ein Mal pro Jahr ☐

Ein bis drei Mal pro Jahr ☐

Öfter als drei Mal pro Jahr ☐

**L3. Haben Sie Zugang zu einem Privatgarten in dem Sie essbare Wildpflanzen sammeln könnten?**

Ja ☐

Nein ☐

**L4. Wie viele Jahre Ihres Lebens haben Sie bisher in Wien gelebt?**

☐☐

**L5. Welche Postleitzahl hat Ihre Wohnadresse?**

☐☐☐☐

LimeSurvey

**L6. Was ist Ihre höchste abgeschlossene Ausbildung?**

- Pflichtschule ☐
- Lehre ☐
- Berufsbildende mittlere Schule (BMS) ☐
- Allgemein bildende / Berufsbildende höhere Schule (AHS/BHS) ☐
- Hochschule / Akademie / Kolleg ☐

**L7. Was ist Ihr Geburtsjahr?**

☐☐☐☐

**L8. Was ist Ihr Geschlecht?**

- Männlich ☐
- Weiblich ☐
- Sonstiges ☐

Sonstiges

**L9. Gibt es abschließend etwas, das Sie uns mitteilen möchten?**
